# Nano/microparticles in conjunction with microalgae extract as novel insecticides against Mealworm beetles, *Tenebrio molitor*

**DOI:** 10.1038/s41598-021-96426-0

**Published:** 2021-08-24

**Authors:** Ivan Rankic, Radim Zelinka, Andrea Ridoskova, Milica Gagic, Pavlina Pelcova, Dalibor Huska

**Affiliations:** 1 Department of Chemistry and Biochemistry, Faculty of AgriSciences, Mendel University in Brno, Zemedelska 1, 613 00 Brno, Czech Republic; 2 Central European Institute of Technology, Brno University of Technology, Purkynova 123, 612 00 Brno, Czech Republic; 3 Central European Institute of Technology, Mendel University in Brno, Zemedelska 1, 613 00 Brno, Czech Republic

**Keywords:** Agroecology, Nanotoxicology, Methods of toxicology studies, Environmental impact, Invasive species

## Abstract

The intensive use of insecticides in global agricultural production has attracted much attention due to its many adverse effects on human health and the environment. In recent years, the utilization of nanotechnology has emerged as a tool to overcome these adverse effects. The aim of this work was to test different microparticles (zinc oxide (ZnO MPs) and silicon dioxide microparticles (SiO_2_ MPs)), and silver nanoparticles (Ag NPs) and to study their toxicity on a model organism, *Tenebrio molitor*. A comprehensive comparative study, which included more than a thousand mealworms divided into nine separate groups, was conducted. In addition to pure nano/microparticle solutions, the effect of particles mixed with the microalgae extract *Chlamydomonas reinhardtii* was also observed. Pure Ag NPs and SiO_2_ MPs resulted in larval mortality of more than 70% compared to that of pure ZnO MPs, in which the mortality rate was approximately 33%. A mixture of the algal extract with zinc oxide microparticles resulted in mortality that was double compared to that observed with pure ZnO MPs. In parallel, atomic absorption spectrometry (AAS) was used to determine the difference in the concentration of trace elements in the bodies of dead and live larvae.

## Introduction

The rise in the global population has consequently increased food demands and caused the global agricultural yields to rise as well as the need for more effective tactics to optimize agricultural strategies mainly against biotic stresses factors^1^. In today's agriculture, the use of pesticides is still unavoidable in spite of their deleterious effects on human health and the environment. Unmanaged and excessive use of pesticides causes various problems as it contaminates our ecological systems, including waterways, sediments and soil, and through transfer of residues across the food chain^2^. However, there is the urgency to find new products that will be more effective, more specific, and less toxic to the environment. The use of GMO plants is currently still debatable and so far, very little supported by the European Union. Therefore, other ways to achieve sustainable agriculture are being sought. Nanotechnology, mainly nanoparticles are intensively studied materials in medicine, material industry, cosmetics and currently also in the agriculture^3^. In recent years, it has been reported that different nano/microparticles (NPs/MPs) have numerous properties that are used for different applications in agriculture, such as fertilizers or pesticides. Several groups have reported both beneficial and/or deleterious effects of different nano/microparticles on seed germination, root elongation and seedling growth^4–6^. In the initial stage of development, the potential of nanoparticles to be used as novel pesticides has already been explored. Evidence showing their toxic effect against selected pests has been detected in most cases along with limited effect on nontarget species. However, a lack of knowledge about possible human, and environmental health implications hinders their practical application and limits their numerous advantages^7^.The broad spectrum of antifungal/antibacterial properties of selected nano/micromaterials has already been reported, but there is little information about their potential as insecticides^8–11^.

The main idea behind our work was to show that nano/microparticles have the potential to provide effective solutions and to assist in novel insecticide creation with increased insecticidal activity and less permanence in the environment. In this study commercially available zinc oxide nanoparticle (after our using—microparticles, ZnO MPs), silicon dioxide nanoparticle (after our using – microparticles, SiO_2_ MPs), and silver nanoparticles (Ag NPs) and their efficiency on the the sixteenth larval stage of the mealworm beetle *(T. molitor)* was researched. The *T. molitor*, is the most important grain product storage pest throughout the world^15^. The control of stored grain pests relies mostly on the broad action of insecticides^16^. Silica is a major component of agricultural soil, and zinc is an essential micronutrient for plant growth. Zinc is widely distributed in plant tissues and is involved in many metabolic processes^12^. Its deficiency reduces growth, tolerance to stress and chlorophyll synthesis^13^. Silver is nonessential for plants, but it stimulates plant productivity at low doses^14^. We compared the mortality rate during the application of ZnO MPs, SiO_2_ MPs, Ag NPs, and an extract of the algae *Chlamydomonas reinharditii* and their effect together with particles against the *T. molitor*. Algae *C. reinharditii* belongs to unicellular flagellates and has been described to produce extracellular metabolites (1-tetradecene, phenol, 2,4-di-tert-butyl-, 1-pentadecene, 1-octadecene, 1-nonadecene, etc.) with antibacterial, antioxidant and anticancer activities, which have been demonstrated by several studies^16–19^. Moreover, algae are becoming attractive to be used in production of biopesticides. Algae and their bioactive compounds have already been used in studies against fungi, bacteria, or insect pathogens in plants such as corn, sunflower, potato, tomato, or watermelon. Such compounds are mainly from bromophenolic, polyphenolic, alkaloids or terpenoid metabolism. Currently, the specific compounds involved are being intensively studied.

## Materials and methods

### Nanoparticles

Commercial zinc oxide nanoparticles (SkySpring Nanomaterials, Inc., Houston, USA, 20–30 nm), silicon dioxide nanoparticles (SiO_2_ NPs, Sigma-Aldrich®, 10–20 nm) and silver nanoparticles (Ag NPs, US Research Nanomaterials, 10 nm) were obtained in powder form. The powders, as received, were dispersed in water (20 g/L), sonicated for ten minutes, and diluted to the desired concentration.

### Characterization of nanoparticles by scanning electron microscopy (SEM)

The samples were dispersed in solution and diluted with demineralized water. Then, the samples were applied to silicon wafers from Siegert Wafer Company and allowed to dry at laboratory temperature (23 °C). This wafer was adhered by a carbon conductive tape to the stub that was inserted into the SEM. The samples were examined by SEM on a Tescan MAIA 3 equipped with an FEG (Tescan Ltd., Brno, Czech Republic). The images were recorded using the In-Lens SE detector at a working distance between 2.92 and 2.99 mm at a 5 kV acceleration voltage under high vacuum conditions. The 768 × 858-pixel images were obtained at 100,000-fold magnification covering a sample area of 2.08 µm. Full frame capture was performed in Ultra Hight (UH) Resolution mode and image shift correction was enabled with accumulation of images, and it took approximately 0.5 min with an ∼0.32 µs/pixel dwell time. The spot size was set at 2.4 nm. The size of the nanoparticles was confirmed by a dynamic light scattering technique (Malvern Instrument Ltd, UK). Zn NPs and SiO_2_ NPs were sonicated in distilled water. After this treatment, the larger NPs aggregated into microparticles.

### Cultivation of algae *Chlamydomonas reinhardtii* and extract preparation

The algae were cultivated under sterile conditions in an Erlenmeyer flask with Tris–acetate-phosphate (TAP) liquid medium at 22 ± 1 °C and illuminated with 130 μmol m^−2^ s^−1^ with a 12 h light/12 h dark photoperiod. After seven days of cultivation, *C. reinhardtii* was lyophilized for 24 h (Feezone 2.5 freeze dryers, LABSCONCO), and 500 mg biomass was dissolved in 200 mL distilled water. Afterwards, the solution was heated at 100 °C and sonicated for 10 min in an ultrasonic bath (K-5 LM, Kraintek s.r.o.).

### Preparation of working solutions

The working solutions of ordered nano/microparticles were made in distilled water or by mixing with algae extract. Additionally, adjuvant silwet star surfactant (SS) was added to each suspension because it facilitates penetration of nano/microparticles across wax substructures^20^. Water lacking nanoparticles was used as a blank treatment.

- To prepare the ZnO MP working solution, 5 mL of ZnO MP stock solution (20 g/L) was dissolved in 45 mL distilled water to obtain a final concentration of 2 g/L MPs. Subsequently, 50 µL of SS was added.
- ZnO MPs + algae extract was prepared by mixing 5 mL of ZnO MPs (20 g/L), 45 mL of *C. reinhardtii* extract and 50 µL SS.
- To prepare working solutions of silicon dioxide microparticles, 2.5 mL (20 g/L) of the SiO_2_ microparticles was mixed with 47.5 mL of distilled water or alga extract and 50 µL SS to obtain a desired concentration of 1 g/L of microparticles.
- Solutions of silver nanoparticles in water or in algae extract were prepared by mixing 1.25 mL of Ag NP stock solution (20 g/L) and 48.75 mL of water or extract. The final silver these suspensions were of 0.5 g/L.

### Cultivation of larvae *Tenebrio molitor*

Larvae *Tenebrio molitor* were bought in an animal shop and were cultured and fed routinely under laboratory conditions (25 ± 2 °C) in Petri dishes (⌀ 90 mm). During the experiments, larvae were divided into nine separate groups, each group containing 150 mealworms (3 P. dishes with 50 mealworms). The larvae were then sprayed directly with prepared NP/MP solutions (approximately 170 µL for each spraying) for five days, and the mortality rate of mealworms was registered every 24 h until the fifth day. Prior to the treatment, the solution was sonicated for five minutes and sprayed. The mortality percentages of *T. molitor* were calculated by using Henderson-Tilton's formula^21^. Dead and live larvae were collected and washed three times with distilled water and subsequently lyophilized for 48 h (Feezone 2.5 freeze dryers, LABSCONCO).

### Sample preparation for AAS analysis

Then, 0.2 g of lyophilized mealworms was weighed into the digestion vessels. The digestion mixture (10 ml of 63% supra pure HNO_3_ diluted by Milli-Q water (Merck, Millipore) in a ratio 1:1 (v/v)) was added to 0.2 g of lyophilized mealworms. The samples were digested by an Ethos One microwave digestion system (Milestone, Italy) at 210 °C for 30 min. After digestion, the samples were stored in the dark in plastic tubes at 4 °C.

Concentrations of Zn and Ag in digested mealworm samples were determined by a 240FS Agilent Technologies atomic absorption spectrometer (Agilent, Santa Clara, USA) with flame atomization and with deuterium background correction. The instrument operated under conditions recommended by the manufacturer with an air-acetylene flame (flow rate 13.5 L/min and 2.0 L/min) and using an ultrasensitive hollow cathode lamp (Agilent Technologies, Santa Clara, CA, USA) as the radiation source of Zn (213.9 nm) and Ag (328.1 nm).

### Statistical analysis

The contents of Zn MPs and Ag NPs in the AAS analysis are expressed as the mean relative standard deviation (RSD) in the Microsoft Excel program and R version 4.0.4. R Core Team (2020). R: A language and environment for statistical computing. R Foundation for Statistical Computing, Vienna, Austria. URL https://www.R-project.org/.

## Results and discussion

In recent years, the use of common fumigants and insecticides to control stored pests in the grain has led to insect resistance^22^. At the same time, these insecticides are also toxic to animals that are fed with grain^23^. Insecticide residues are then transferred to the animals and then to humans, so an alternative strategy to protect stored grains needs to be found. Nanotechnology and nanoparticles are now entering the field of agricultural biotechnology^24,25^. Slowed agrochemical release kinetics and reduced application volume are the main advantages of nanoparticles^26^. Recently, there has been growing interest in research into NPs and NPs as potential insecticides. Nanoparticles (NPs) have been tested against insect pests from different order like as *Coleoptera*, *Lepidoptera*, *Hemiptera*, *Diptera*, such as silver (Ag), gold (Au), aluminum (Al), silica (Si), and zinc (Zn) and metal oxide, zinc oxide (ZnO) and titanium dioxide (TiO_2_)^22,27–33^.

**Figure 1:**
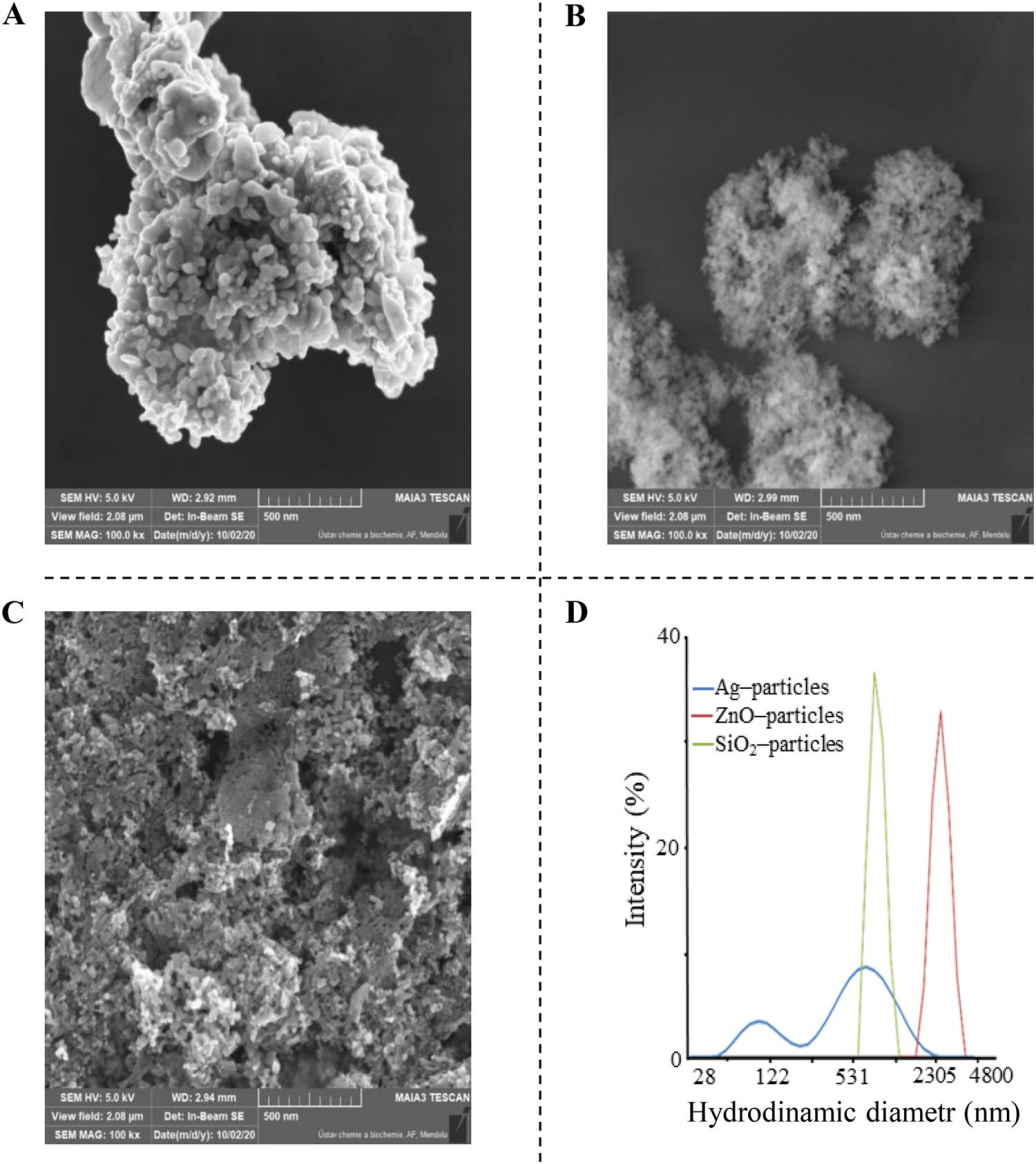
SEM images of **(A)** Ag NPs, **(B)** SiO_2_ MPs, and **(C)** ZnO MPs. **(D)** DLS particle size distribution profiles of nanoparticles in aqueous solution.

To determine the size and shape of the nanoparticles, SEM was used. Figure 1 shows that the average diameter of nanoparticles in solution after sonication was larger than that stated by the manufacturers. The microscope images indicated that the morphology of the particles was spherical in shape and consisted of aggregates several micrometres in diameter, as seen in the images (Fig. 1A,B,C). The SEM micrographs of Ag NPs showed that aggregates consist of fine structured particles of various sizes. This was also confirmed by dynamic light scattering (DLS) analysis. The same results were obtained using Zn nanoparticles and SiO_2_ nanoparticles. The obtained results show their differential distribution profiles, which are consistent with the SEM results (Fig. 1D). The size of particles using DLS analysis showed an average size of 530 nm for Ag NPs and 777 nm for SiO_2_ MPs and ZnO MPs (2355 nm). Additionally, the surface charge and colloidal stability of the used nanoparticles were determined by analysis of the zeta potential. Nanoparticles dispersed in water showed zeta potentials of −24.2 mV, −30.2, and −28.7 mV for Ag NPs, SiO_2_ MPs, and ZnO MPs, respectively. These values are slightly below the threshold of -30 mV, which is considered the minimum zeta potential for electrostatically stabilized suspensions^34^.

**Figure 2:**
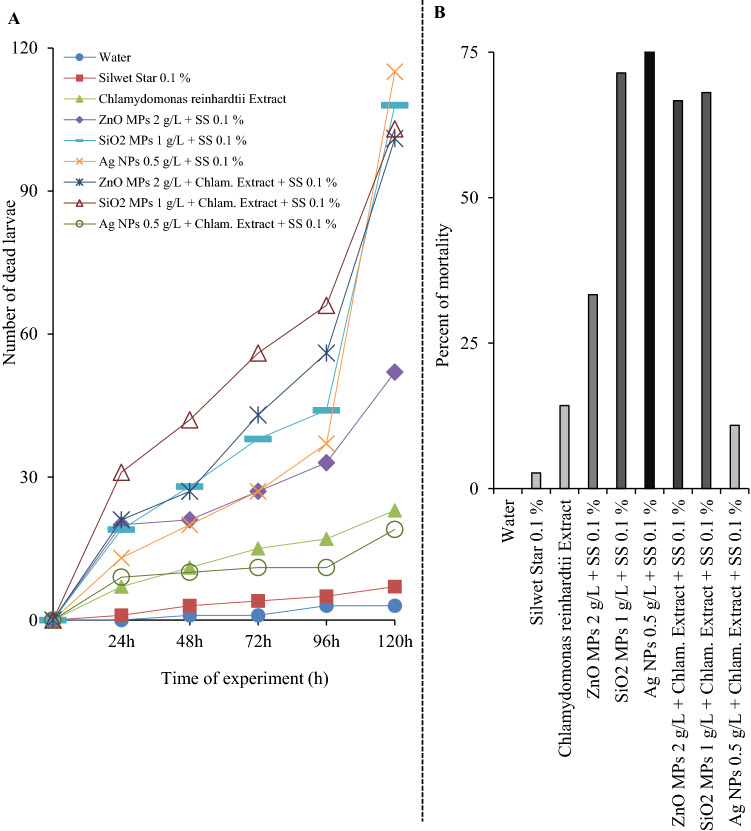
Mortality of *Tenebrio molitor* larvae after using pure 2 g/L ZnO MPs, 1 g/L SiO_2_ MPs and 0.5 g/L Ag NPs and these MPs/NPs in combination with *Chlamydomonas reinhardtii* extract. **(A)** Number of dead larvae for 5 days. Three spray treatments: 0, 48 h, 96 h. 150 mealworms were used per treatment. **(B)** Percent of total mortality after 120 h. Mortality was measured by the Henderson-Tilton formula^20^.

This study was devoted to investigating the effect of NPs/MPs alone and NPs/MPs with algae extract on the viability of the sixteenth larval stage of *T. molitor* and to examining their insecticidal effect. The efficiency of selected nano/microparticles (ZnO MPs, SiO_2_ MPs and Ag NPs) against *T. molitor* was tested at concentrations of 2 g/L, 1 g/L, and 0.5 g/L and is presented in Fig. 2. Mortality was monitored for five days, and the results were collected every 24 h. Selected concentrations of NPs/MPs were selected based on preliminary experiments.

Our results indicate that *C. reinhardtii* extract had a negligible activity when tested alone (14%), while an increase in mortality was observed following treatment with ZnO MPs in water and when mixed with algae extract. The ZnO MP-treated larval mortality was 33%, while the mortality when mixed with *C. reinhardtii* was 66%. Recent investigations of *C. reinhardtii* have shown its antioxidant activity and antibacterial potential against different strains^16^. Such activity is assumed to be related to its major extracellular metabolites.

Indeed, 1-nonadecene, 1-octadecene, 1-tetradecene, and diisooctyl phthalate have been shown to exert insecticidal or antibacterial activity across several species^35^. Although the mechanism of *the C. reinhardtii* extract was not addressed in detail here, the increased larvicidal activity of nanoparticles when mixed with its extract might be derived from the actions of bioactive compounds such as phenolic compounds and flavonoids^16,36^. Previous studies on the antimicrobial activity of ZnO nanoparticles hypothesized that reactive oxygen species (ROS) generated by the surface of ZnO NPs triggered irreversible damage to the microbial cell wall. Together with active molecules from algae extract can result in compromised cellular integrity and eventual pathogen death.

From Fig. 2, differences were obvious between different nanoparticle treatments; however, this was expected because the effect of NPs/MPs appears more complex when linked with factors such as composition, shape, size, and surface-to-volume ratio^37^. Different mechanisms of nanoparticle action may be a main reason for the observed difference in activities. In our experiment, silver nanoparticles were the most effective against mealworms. The larval effect of the 0.5 g/L Ag NPs on the larvae was confirmed compared to the untreated control. It is still unclear what makes Ag NPs less effective when they are combined with algae extract. One of the hypothesis could be the fact—that metabolites from the algae extract have bound to the surface of the NPs and thus inactivated the activity of the silver NPs. However, this statement needs to be investigated in more detail in a further experiment. Silver has been employed commonly as a component of many plant antimicrobial formulations.

The toxic nature of silver ions is well known, and one of the possible mechanisms that has been reported relies on the ability of silver ions to bind to cysteine-containing proteins, causing pathogen membrane disruption^38^.

Many studies have described the insecticidal effect of silica nanoparticles^31,39–41^. The reported underlying mode of action is through desiccation of the insect cuticle after nanoparticle absorption to the cuticular lipid and disruption of the structures^42^. The exposure of *T. molitor* larvae to silica microparticles inflicted a mortality rate of approximately 71% and resulted in a darker cuticle of dead larvae. We confirmed the hypothesis that SiO_2_ NPs damage epidermal and dermal cells, which leads to dehydration of the larval body, making them look dark^39^. This morphological alteration in the presence of silica MPs coincided with the declines in cell viability^43^ and may be, thus, assumed to be associated with larval death by desiccation.

Table 1 shows the statistical analysis of *T. molitor* mortality expressed as the mean ± SD with Tukey HSD inference. All treatments were compared with a control sample (treated with water). The sample treated with 0.1% Silwet Star surfactant was insignificant. Pure *C. reinhardtii* extract showed statistical significance (*p < 0.05). Other samples showed a significant difference (** p < 0.01) compared with that of the control sample.

**Table 1:** Statistical analysis of *T. molitor* mortality expressed as the mean ± SD with Tukey HSD inference.

Treatment	(mean ± SD)	Statistical analysis of mortality
		Tukey HSD inference
Water	1.00 ± 0.00	/
Silwet Star 0.1%	2.33 ± 0.58	insignificant
*Chlamydomonas reinhardtii* Extract	7.67 ± 1.15*	p < 0.05
ZnO MPs 2 g/L + SS 0.1%	17.33 ± 0.58**	p < 0.01
SiO_2_ MPs 1 g/L + SS 0.1%	36.00 ± 3.61**	p < 0.01
Ag NPs 0.5 g/L + SS 0.1%	38.33 ± 2.31**	p < 0.01
ZnO MPs 2 g/L + Chlam. Extract + SS 0.1%	33.68 ± 3.79**	p < 0.01
SiO_2_ MPs 1 g/L + Chlam. Extract + SS 0.1%	34.33 ± 0.58**	p < 0.01
Ag NPs 0.5 g/L + Chlam. Extract + SS 0.1%	6.33 ± 0.58**	p < 0.01

Similarly, compared to their bulk materials, silicon, titanium dioxide, copper, alumina, zinc, and silver nanoparticles have emerged as potential candidates for combating different agricultural pests, improving plant responses to various biotic and abiotic stresses, and enhancing plant growth performance^44–46^. Additionally, there may be numerous mechanisms of nanoparticle toxicity toward insect pests. Certain nanoparticles can penetrate and accumulate in the cell membrane, which is likely to cause cell lysis, while others may stimulate the generation of cellular ROS, leading to loss of cellular function and cell death^47,48^.

The accumulation of ZnO MPs and Ag NPs was estimated by atomic absorption spectroscopy (AAS). For technical reasons, we could not measure the accumulation (content) of silicon in the bodies of *T. molitor* larvae. The zinc and silver concentrations in live and dead larvae (Table 2) were expressed in units of mg/kg. The zinc content was determined in all tested larvae (Table 2). However, in larvae treated with ZnO MPs, a higher zinc concentration was observed compared to that of larvae not treated with ZnO MPs. The highest values of zinc (268.01 ± 9.20) were measured in dead larvae sprayed with a solution of ZnO MPs at 2 g/L with algal extract and in live larval bodies (225.41 ± 6.20) sprayed with the same solution. The increase in zinc concentration in both dead/live larvae reveals that these MPs were able cross larval cuticles when mixed with the algae extract. The water-sprayed control sample contained significantly p < 0.001 less 48.02 ± 4.60 Zn in live larvae; in contrast, 70.00 ± 9.60 mg/kg zinc was measured in dead larvae.

**Table 2:** The total content of Zn and Ag ions in bodies of live and dead larvae measured by atomic absorption spectrometry (AAS) with flame atomization and with air-acetylene flame and using ultrasensitive hollow cathode lamp as the radiation source of Zn (213.9 nm) and Ag (328.1 nm). The data were statistically analyzed (dead to live larvae) by one-way ANOVA variance test with subsequent Tukey's multiple comparison test. The asterisks represent statistically significant differences (* = p < 0.05, ** = p < 0.05).

Treatment	live larvae	dead larvae	live larvae	dead larvae
	Zn ± RSD	Zn ± RSD	Ag ± RSD	Ag ± RSD
Water	48.02 ± 4.60	70.00 ± 9.60**	0.00 ± 0.00	0.00 ± 0.00
Silwet Star (SS) 0.1%	47.19 ± 8.90	90.67 ± 3.20**	0.00 ± 0.00	0.00 ± 0.00
*Chlamydomonas reinhardtii*-extract	44.60 ± 9.20	83.33 ± 8.50**	0.00 ± 0.00	0.00 ± 0.00
ZnO MPs 2 g/L + SS 0.1%	97.57 ± 5.20	120.77 ± 6.40**	0.00 ± 0.00	0.00 ± 0.00
Ag NPs 0.5 g/L + SS 0.1%	48.82 ± 5.80	52.38 ± 5.80	6.02 ± 4.30	12.89 ± 4.80*
ZnO MPs 2 g/L + Chlam. Extract + SS 0.1%	225.41 ± 6.20	268.01 ± 9.20**	0.00 ± 0.00	0.00 ± 0.00
Ag NPs 0.5 g/L + Chlam. Extract + SS 0.1%	44.54 ± 4.50	80.89 ± 3.40**	12.24 ± 5.90	16.54 ± 6.90

All arthropod groups contained high concentrations of zinc, iron, manganese and other microelements and heavy metals in their bodies. These metals are found as part of the cuticular components of the body^49,^^50,51^. Zinc is an essential component of more than 300 enzymes and transcription factors. In addition to the cuticular part of the body and the enzyme complex, zinc also plays a role in DNA synthesis and is essential for the proper physiological functioning of insects, and it is also found in the area surrounding the midgut (including Malpigh tubules) in pupae and adults^52,53^. To maintain homeostasis and reduce toxicity, the content of micronutrients is regulated. The rate of zinc excretion does not exceed the rate of accumulation until it is near toxic levels, and thus more zinc accumulates than necessary. Mir et al.^54^ studied the accumulation of zinc in the whole body of the *Bombyx mori* insect, which was fed a leaf treated with ZnO NPs. During their experiment, they found that zinc had accumulated in the body for 6 h, after which time its levels began to decline.

Table 2 also shows the total content of silver nanoparticles (Ag NPs). Silver was detected only in the larvae that were subjected to Ag NPs with or without algae extract. The highest content of Ag NPs was measured in the dead body of mealworms treated with Ag NPs and algal extract (16.54 ± 6.90 mg/kg). In contrast, the lowest measured silver content (6.02 ± 4.30) was recorded in the bodies of live larvae treated with pure Ag NPs. Similar values were measured in the live body of mealworms (12.24 ± 5.90) treated with Ag NPs and *C. reinhardtii* extract and dead larvae treated with pure Ag NPs (12.89 ± 4.80). Silver was not detectable in the other samples because silver is not an essential micronutrient in living organisms. Although silver, which is not part of cells and tissue, has good antimicrobial effects and insecticidal effects that induce cytotoxicity, increase ROS production, and lead to DNA damage and apoptosis^55,56^. Ionic silver strongly interacts with thiol groups and deactivates vital enzymes^57,58^. At present, relatively high attention has been given to silver nanoparticles. Various studies have shown that Ag NPs are able to distribute and accumulate in certain organs of the body after exposure^59,60^. Silver nanoparticles have effects on the development and growth of larvae, the duration of larval and pupal stages and the viability of adults. It was found that the amount of Ag NPs increased in *Drosophila melanogaster* as the dose of exposure increased, and insects were able to accumulate silver in the tissue for a long time even when the organisms were not exposed. Further studies have also found that Ag NPs accumulate in the body after application, lead to demelanization of the body and have an insecticidal effect on the model organism *D. melanogaster*^61^.

## Conclusion

Novel tools for the management and control of insect pests with agricultural importance are needed. In certain cases, inorganic, pristine nanoparticles, which are not intentionally produced for pesticidal applications, such as the metal/metal oxide form of the nanoparticles, may trigger this biological effect^62,63^. Herein, we evaluated the larvicidal potential of three different types of commercially available nanoparticles on the survival of the sixteenth larval stage *Tenebrio molitor*. Our results showed that pure silver NPs and silicon dioxide microparticles had insecticidal effects of more than 70% on larval viability. Additionally, we explored the efficiency of nano/microparticles in the presence of the extract of algae *Chlamydomonas reinhardtii* Algal extract in combination with ZnO MPs increased larval mortality to twice (66%) compared to that of pure ZnO MPs (33%). In contrast, algal extract reduced the effectiveness of silver nanoparticles and mortality decreased to 11% compared to pure Ag NPs (76%). Components of algae extracts are considered safe for human health and the environment, and herein, it is shown that these active molecules may offer solutions and strategies for controlling insect pests by improving the insecticidal effects.

### Ethical approval

This article does not contain any studies with human participants or animals performed by any of the authors.

### Declaration of authors

The final manuscript has been read and approved by all named authors. We warrant the originality of the presented work, which is not under consideration for publication elsewhere.
